# Contrasting staff and student views on alcohol education provision in a UK university

**DOI:** 10.1080/09687637.2018.1475548

**Published:** 2018-05-29

**Authors:** Rachel Brown, Simon Murphy

**Affiliations:** DECIPHer, School of Social Sciences, Cardiff University, Cardiff, UK

**Keywords:** Alcohol education, university/college, qualitative, harm reduction

## Abstract

Alcohol education and awareness aims to teach individuals the risks of excess consumption. It is common in UK universities, despite limited evidence of success with student cohorts. This research explored the development and delivery of such alcohol activities at one UK university. In-depth, one-to-one interviews were carried out with non-academic staff and with first-year students. These aimed to understand the development of alcohol awareness messages and staff involvement in delivery, as well as exploring student responses to key alcohol educational activities. Results indicate that alcohol is a normalized aspect of UK student identity and is accepted as such by students and staff. Despite this, there is a widely held view that the university has a responsibility to provide alcohol education and awareness, which forms the basis of current practice on campus. This reflects perception of education interventions as non-coercive and acceptable within the staff–student relationship, with limited support for more interventionist approaches with a group engaging in a legal behavior with strong cultural associations. However, staff approval of education as appropriate for this audience is contradicted by students, who reject these same approaches as reminiscent of school, instead favoring self-directed learning or peer-led programs.

## Introduction

UK university settings are perceived by students as permissive of heavy alcohol use (Morton & Tighe, 2011), and high-risk drinking has been evident in UK student populations for many years (Gill, 2002). This is frequently characterised by heavy, single occasion – or binge – drinking (Craigs, Bewick, Gill, O’May, & Radley, 2011; Morton & Tighe, 2011), and has been linked to multiple adverse health and behavioral outcomes, including accidents (Clapp, Shillington, & Segars, 2000), being a victim of crime (Newbury-Birch et al., 2009), and increased risk of unprotected sex (White & Hingson, 2013). Although current data suggest a drop in overall consumption among UK young adults, it is as yet unclear whether this decrease is translating into reduced alcohol-related harms in youth populations, including students (Institute of Alcohol Studies, 2013). As such, the most recent UK Government alcohol strategy states continuing expectations that universities will work to educate students on the risks associated with heavy consumption (HM Government, 2012).

Many UK universities undertake alcohol education and awareness work, traditionally based on empowerment models of health promotion, meaning provision of information to individuals to inform different choices (Dunne & Somerset, 2004). Despite little evidence of a knowledge deficit in young drinkers in relation to alcohol harms (IAS, 2013), typical activities include display of safe drinking messages warning of potential consequences of drinking to excess (Orme & Coghill, 2014).

However, delivery of alcohol programs within UK universities is variable (Orme & Coghill, 2014) and often lacking impact (Larimer & Cronce, 2002). Assessment of alcohol education campaigns is hampered by absence of robust evaluative data (Foxcroft & Tsertsvadze, 2012), with programs in university settings often lacking control or comparison groups, having small sample sizes and limited long-term follow up (Barnett & Read, 2005). Where evaluation has occurred, evidence suggests that increased exposure to alcohol education messages does not correlate with decreased binge drinking or reduced harms (Larimer & Cronce, 2002; Wechsler et al., 2002). Alcohol education campaigns have been shown to increase alcohol knowledge in first-year students but not to reduce high-risk drinking compared to controls (Croom et al., 2010). At both university (Cronce & Larimer, 2011) and school level (Anderson, Chisholm, & Fuhr, 2009), alcohol education-only campaigns fail to demonstrate reduction in alcohol-related harms, or fail to sustain effects over time (Paschall, Antin, Ringwalt, & Saltz, 2011).

Despite these limitations, internet searching for ‘alcohol awareness at UK universities’ quickly illustrates that such programs are routinely pursued and continued at campuses across the country. In the absence of evidence of impact on student drinking, it is important to consider why this occurs and to understand organizational decision making underpinning these practices. This can provide insights into barriers to implementation of alternative approaches which may more effectively reduce harms.

This paper presents an in-depth exploration of alcohol practices in one UK university. Case selection was not based on unique institutional features but on opportunity to study the research problem. The case should not be considered as a ‘typical’ example of a university, with concepts of typicality deemed problematic in social settings where each possesses unique processes and interactions (Stake, 2000). However, this acknowledgement of uniqueness does not exclude the possibility of gaining insights that may contribute to discussion of processes at other universities.

The research explores how current campus activities on alcohol education and harm reduction are formulated, which staff are involved and the rationale for these activities. It also examines student responses to awareness-raising messages to consider whether they are being received as intended. In doing so it highlights a key contradiction between staff and student views of appropriate action on alcohol and the implications of this for understanding absence of impact.

## Materials and methods

This study explores perceptions of – and contributions to – institutional activities aimed at addressing alcohol related harm. Design involved in-depth, qualitative interviews, which were selected to facilitate exploration of meaning for those central to the enquiry (Bryman, 2008). This paradigm was relevant for understanding student alcohol consumption and its relationship to the complex, multi-level university setting (Dempster, 2011). Interviews involved non-teaching staff and first year students. Ethical approval was obtained from the University Research Ethics Board. The research was conducted at one UK university, located centrally in a mid-sized UK city. The student population is over 25,000 and a majority of first-years live in university-owned residence halls.

### Staff recruitment

The lead researcher has previously delivered alcohol awareness campaigns in universities and was familiar with typical staff involvement in such work, so sampling strategy was initially guided by this. Exploratory phone calls were made to managers across a range of university departments, including Human Resources, Residential services, Student Support, and also Student Union (SU) to discuss current alcohol activities and who was involved. This identified several additional departments for inclusion. Permission was sought from relevant managers to contact staff to discuss interviews however, for confidentiality, managers were not informed which staff within departments were subsequently approached. Interviews were conducted with 17 staff (eight male) who self-identified as being involved in the development of alcohol policies or awareness activities at the time of interview (Table 1 for full details). Eight interviewees were in managerial roles in their departments and, of these, six were male.

**Table 1.** Staff participants (gender is indicated in brackets).

Interviewee	Department
P1 (M)	Student union
P2 (F)	Student support
P3 (F)	Student counselling
P4 (F)	Campus security
P5 (F)	Residential services
P6A (M)	Residential services
P6B (F)	Residential services
P6C (F)	Residential services
P7 (M)	Occupational health
P8 (M)	Student counselling
P9 (M)	Student health services
P10 (F)	Student support
P11 (M)	Student support
P12 (M)	Student union
P13 (M)	Student registry
P14 (F)	Student counselling
P15 (F)	Student safety partnership

### Student recruitment

Sample drew from students who had moved away from the family home to attend university due to evidence of higher alcohol consumption among this cohort, both in the UK (Thombs et al., 2009; Ward & Gryczynski, 2009) and internationally (Dantzer, Wardle, Fuller, Pampalone, & Steptoe, 2006). This was determined by related study considerations of associations between alcohol and social behavior. University policy prohibited contact with students through emails therefore recruitment was carried out through face-to-face contact outside of residences sites, where the research was outlined to prospective participants and a flyer with study details and researcher contact information was provided. Due to slow response rate, this was then supplemented through direct promotion to whole class groups, utilizing the lead authors part-time teaching work. This direct approach is commonly utilized in organizational studies where the researcher may be in a position to access a population due to their own role (Bryman, 2008). It was essential that students felt no pressure or coercion due to the power differential inherent in the institutional relationship (Miller & Bell, 2002) therefore students were asked only if they would be willing to take study information sheets back to their accommodation to leave visible to flatmates. Several who agreed to this did subsequently request the opportunity to take part themselves and were accepted into the study. Gender parity was aimed at in recruitment but not achieved, with approx. 60% female sample (*N* = 23, nine male). All but two students lived in university-run accommodation, with the other two sharing a private rented house. Brief biographical information on student participants is presented in Table 2.

**Table 2.** Introduction to participating students.

	Personal details	Before arriving	Since arriving
S1	Female, 18 years old	Drank before Uni but mainly just pubs and rarely clubs.	Lives in halls. Made choice after reading about which halls were most ‘lively’. Drank a lot during Freshers and still goes out a lot with flatmates.
S2	Male, 19 years old	Had visited the area before and knew there would be a lively nightlife compared to home.	Lives in halls. Made choice of hall after reading online about costs and location. Drinks a lot, mostly with friends from sports teams.
S3	Male, 18 years old	Often drank at friend’s houses before coming but parents are both non-drinkers so never drank at home.	Lives in halls and chose from reading about the ‘most social’ option. Gets drunk with housemates a ‘couple of times a week’.
S4	Female, 19 years old	Drank before uni but was ‘never too bothered’ about it.	Moderate drinker. Lives in halls. Found Freshers quite tough as a moderate drinker but found a good group of friends and is living with them next year.
S5	Female, 18 years old	Went to pubs quite a bit before uni but was worried about pressure to drink differently at uni.	Moderate drinker. Lives in halls but friends are mainly other occasional drinkers from student societies.
S6	Female, 19 years old	Drinks ‘very rarely’, mostly when going out to listen to music rather than just to drink.	Low/non-drinker. Lives in halls but found it really hard in Freshers due to drinking levels of flatmates.
S7	Female, 19 years old	Visited the area once before starting and really liked it. Drank a bit before uni but was worried about lack of experience.	Low/non-drinker. Lives in halls and has had difficulties because of drinking levels of flatmates.
S8	Female, 20 years old	Drank before Uni but only a couple of local pubs to choose from. Was excited about the local nightlife.	Lives in the ‘social hall’ and her flat is often used for pre-drinks. Binge drinks a few times a week.
S9	Male, 20 years old	Felt like he had plenty of drinking experience during his year out before Uni.	Lives in halls. Picked a ‘social’ hall. Goes out drinking with flatmates at least once a week.
S10	Male, 19 years old	Drank in local pubs before Uni and was looking forward to a bigger range of places to go.	Lives in halls. Drank a lot in Freshers but mostly drinks with course friends now. Binges a couple of times a week.
S11	Female, 18 years old	Didn’t drink much before Uni.	Lives in private housing and mostly goes to local pubs with housemates every week, ‘at least once’.
S12	Male, 20 years old	Had a gap year working and drank a lot during this time.	Lives in halls and drinks a lot, both with flatmates and sports society friends.
S13	Female, 19 years old	Drank before uni but not many options at home so just local pubs.	Chose the biggest hall to meet the most people. Drinks frequently with house mates.
S14	Female, 21 years old	Knew the area well and was looking forward to the nightlife as a student.	Chose the ‘liveliest’ hall and ‘likes a drink’. Goes out multiple times a week to get drunk.
S15	Female, 19 years old	Non-drinker.	Low/non-drinker. Chose one of the smaller halls to avoid so much drinking. Some people have had issues with her not drinking but has found friends now who don’t care.
S16	Male, 20 years old	Drank regularly before uni.	His hall is ‘too quiet’ so he goes to friends’ houses to drink ‘pretty often’. Likes getting drunk.
S17	Male, 19 years old	Drank in local pubs back home but not much clubbing experience.	Lives in halls, has a drinking night with flatmates and another with course mates ‘most weeks’.
S18	Female, 19 years old	Drank moderately before uni, mostly in pubs with friends.	In the biggest hall and happy about the chance to meet lots of people. Gets drunk ‘maybe weekly’.
S19	Female, 18 years old	Hadn’t drunk much before coming but the area had a good reputation for nightlife at home.	Lives in halls. Is a regular drinker and enjoys the local club scene.
S20	Female, 19 years old	Drank a bit in pubs before coming but no clubbing experience.	Lives in a quieter hall so goes to other friends’ houses for drinking, maybe a couple of times a week. Likes ’pre-drinks’.
S21	Male, 19 years old	Didn’t know much about the area but had heard that locals ‘liked a drink’.	Chose the most social hall but got placed in a quieter hall instead. Likes drinking and ‘goes out a lot here’.
S22	Male, 18 years old	Defined as a ‘special occasion’ drinker before uni.	Lives in private housing. Drinks with them once or twice a week.
S23	Male, 19 years old	Enjoyed going to pubs and clubs before uni ‘for the atmosphere’.	Chose the most ‘social’ hall and gets drunk ‘a fair bit with flatmates.

### Data collection: mapping the research context

To inform development of interview schedules, activities were undertaken to identify and map current alcohol education or harm reduction activities at the university. Two site visits were made (with permission) to key areas including SU and Residences. A written record was made of any visible materials containing an awareness-related message, including key content, location and authorship when available. Posters were observed from two UK charities working to raise awareness of alcohol harms. This included messaging from Drinkaware (an alcohol education charity funded by UK alcohol producers, retailers and supermarkets), on the dangers of being too drunk, and from Alcohol Concern, including promotion of the ‘Dry January’ month-long abstinence campaign. Advertized activities also included a forthcoming alcohol awareness week in the SU, and ads for a safe taxi service for drunk students, run in partnership with a local taxi firm. The Union further publicized a policy of refusal of service for drunk patrons. As is the norm, the university also operated a general advice centre, which does not specifically address alcohol issues but offers signposting to specialist outside alcohol support agencies.

A request was also made to Human Resources for access to any policies mentioning alcohol. Drawing on Rapley (2008), these were analyzed to identify: embedded rules on alcohol; target of the text; structure and organization; use of evidence; embedded discourse on alcohol. Searches of the university website also identified two alcohol awareness webpages, which were subsequently discussed in interview, one outlining consequences of drinking on social and psychological function and the other containing tips for reducing drinking on a night out.

From this mapping activity, questions were formulated for topic guides to understand both staff and student awareness and opinions, of identified materials, with staff also asked about any direct involvement in development. Students were asked for their views of identified materials and also about their own typical drinking activity so far. As the research focus was on perceived importance of alcohol during transition and adaptation, students were not asked to quantify consumption but were instead asked to self-define their ‘typical’ drinking. All interviews lasted approximately 45–60 min and took place in a private room where confidentiality could be maintained and all were transcribed in full.

### Data analysis

Open reading was first carried out by the primary author to identify emerging themes for coding. Second-phase coding (Saldaña, 2013) involved thematic analysis to identify and report patterns (Braun & Clarke, 2006), with reference to findings from an earlier literature review to further develop these themes and ensuring an iterative analytical approach. Coding was revised and finalized through discussion between authors. Staff and student transcripts were initially coded as separate data sets, using Word comment and text searching functions, before comparison and examination across the sets to further refine and develop key themes. Quotes are used below to illustrate themes identified through this analysis. Within the paper, staff are referred to as P(n), gender (M or F) and job role included on first mention, and students as S(n) and gender (M or F).

## Results

This section presents findings from analysis of interviews with staff and students, with illustrative quotes throughout. It considers expectations of alcohol in university culture and participant views of appropriate responses. It then considers the types of activities being delivered on campus at the time of data collection and perceptions of these.

### Expectations of alcohol use

Students were asked to describe own drinking as *non/low, moderate, regular drinker,* with no quantification offered in order to capture self-perception. Three of those interviewed identified as low/non-drinkers, two as moderate drinkers and the remainder self-defining as drinking regularly. Moderate and regular drinkers were asked whether consumption involved binge drinking, with a majority interpreting this as meaning drinking to a point of drunkenness. Most described this as being the norm during Freshers, with a typical decrease to once or twice weekly (for regular drinkers) and less often for moderate consumers. Interestingly, moderate consumption was interpreted as getting drunk less often rather than consuming less on drinking occasions. Drinking in-between binges was rare for this group. Of three students who defined as low/non-drinkers, two of these discussed light drinking at social gatherings as a way of joining in, while the other was abstinent.

When asked what role alcohol plays in student life, a large majority positioned it a traditional part of student identity. This was expected by both drinkers and low/non-drinkers as being what ‘most’ students would do:

I don’t know where the whole drinking craze came from at university but obviously the two are entwined and because of that people must just sort of shake off all the thoughts about what alcohol does to you, because you feel like you go to university you’re going to have to drink, the two are like inseparable… (S15, F)

I guess it (drinking) is sort of like a traditional way of getting to know people. When I say traditional I mean traditional like the student sense and not the goes back through the ages. (S16, M)

Reflecting inter-generational normalization of alcohol in student culture, staff also reflected this view and made frequent references to own drinking experiences at university:

I think particularly within British society there is that expectation that when you go to university you’re going to be drinking excessively, you’re going to be using drugs, you’re going to be partying. And that seems to be quite fixed within our culture. I mean it was the same when I went to university 20 odd years ago. (P3, F, Student Counsellor)

Among staff, this acceptance was frequently accompanied by reflections on alcohol as part of the rite of passage for young adults moving away from home and commencing the journey to independence, for example:

…they’re coming to cut the ties from their parents and to become adults in their own right and make their decisions, whether it’s a good or bad decision, whether its falling over drunk and friends helping them and put them to bed…not necessarily that we want to encourage but that can be part of the university and its them making decisions as an adult and learning what it is right from wrong. (P2, F, Student Support Advisor)

To understand the manifestation of this view in alcohol practices, we discussed what the role of the university should be in relation to student drinking.

### Staff views of appropriate action

Staff were asked that they thought the university should do about student drinking and, while most felt that there was limited chance of reducing consumption, it was commonly accepted that there was a duty to do something. What this should be was complicated by varying views of what the university should represent in the lives of students.

For staff, there was an obvious challenge between having a duty of care for student well-being, with understanding that alcohol could compromise that, and over-stepping boundaries of an appropriate role for the university. Most felt that university approaches to alcohol should reflect positioning as an educational body, for example:

I think there is an issue about where the duty of care for our students begins and ends. What we mustn’t do ever, is act as some sort of encouragement to drink more than you’re capable of drinking…we’ve all got to keep educating students about sensible limits. (P13, M, Student Registrar)

We explored perceptions of more regulatory approaches identified from the research literature and presented as examples of practice elsewhere, such as ‘dry’ halls of residence, limitations to on-site sales, fines for alcohol-related behavioral issues such as noise, etc. Staff in Residential settings felt that the landlord–tenant relationship with students meant limited right to moderate behavior through more interventionist measures:

They’re sort of customers you know they’re not naughty people, they pay us to live here. Your landlord doesn’t come round to yours and say “only one bottle of wine a week” you know it is very difficult. There’s a line and you can quite easily cross it. (P6B, F, Residences officer)

Others suggested that the ‘student-as-customer’ status created by the current fee-paying structure created further constraints to actions deemed appropriate:

If I’m paying nine grand I don’t know that I would want someone to be telling me how I should live my life. I think health promotion is a kind of take it or leave it isn’t it. …I’m not sure that people should necessarily be disciplined by the university if they happen to end up in A & E (Emergency Room) after a night out on the lash. (P8, M, Counselling Manager)

Education and awareness raising, specifically on the consequences of excess consumption, were widely favored over such policy-led approaches. This educational focus was deemed consistent with both the pedagogical role of the institution and the right of students as adults:

…we do what we can…it’s a very difficult balance because students, for the vast majority, they’re 18 plus. They’re adults and whilst we can educate and promote health and well-being, people will want to live independently, and for many it’s the first chance they’ve had to do that. (P7, M, Deputy Manager, Occ. Health)

I keep harping on about the educational side of it as opposed to the directive side. This is not a school, this is a university, you’re dealing with adults. As long as we are, I think, giving students the right advice, the right support, then, then that probably is sufficient. (P13, M)

### Staff involvement in policy and practice

As no standalone alcohol policy for students was in place at the time of data collection, staff were asked whether they would favor having a policy and what could be achieved through this. Responses on the utility of this were highly variable, with observations that staff were limited by lack of capacity to enforce content. It was further suggested that alcohol policy may exceed reasonable standards of duty of care and stray into attempts to ‘*micro-manage students’ lives*’ (P13).

Alcohol was discussed within the existing general disciplinary policy which governed behavior in communal areas and damage to property. This reflected a behavior management approach, and was developed – and utilized – predominantly by Residences staff, based on the greater significance of alcohol to their daily working:

When it’s been a possible disciplinary issue in terms of damage…99 times out of a hundred, alcohol is involved somewhere with the damage (to property). (P5, Residential Manager)

Residences staff, and particularly P5, also led on the content of the alcohol webpages described earlier, which were developed internally as a response to the frequency of alcohol issues in halls and the perception of alcohol awareness and education as the best approach.

As well as Residences, the SU was identified as the other main location for alcohol policy and awareness activities. They also utilized a disciplinary approach to alcohol behavior management, implementing premises bans for unacceptable behavior, and it was evident that the SU was primarily conceived of as a responsible retailer and a trusted licensed premises:

We’re not like your average bar or nightclub environment so we’re not solely in it to make as much money as we can…there’s a commercial income stream but we’ve got a duty of care to our members…it is just taken a bit more seriously than it would be in a nightclub in town for example. (P1, M, SU Manager)

This duty of care manifested in awareness and educational activities, including poster campaigns and alcohol awareness week events described earlier. Most other staff suggested that it was right that the SU, as the predominant alcohol retailer on site, should have primary responsibility for carrying out alcohol awareness activities. However, this was often coupled with suggestions that this was unlikely to be prioritized where alcohol sales were financially beneficial to the SU, reflecting arguments that where competing economic and health interests are observed, economic concerns will win (Jayne, Valentine, & Holloway, 2008):

The events they run give a contradictory message to those from elsewhere because the SU needs to make money…Nights like Drink the Bar Dry. The duty of care differs between the university and the union – their primary role is different… (P16, F, Security Co-ordinator)

In terms of effectiveness, the delivery context within the bar/nightclub setting is likely to be significant, as evidence suggests that such messages may be seen as either already known or contradictory to the aims of attending such a drinking space (Brooks, 2011). It is notable that staff generally expected the impact of these education and awareness messages to be low, specifically with the ‘Dry January’ campaign likely to be limited by timing and very low numbers of students around campus in January exam periods, meaning limited exposure to the messaging on display.

### Student views of current practice

Staff descriptions and mapping of campus activities informed interviews with students, with questions on expectations of any university rules on alcohol, and opinions of educational information currently available. All students, regardless of drinker status, rejected the appropriateness of the university setting any rules aimed at limiting consumption, citing legal adulthood as the primary reason for this:

We’re all over 18 so I wouldn’t see why there would be (rules on alcohol). (S1, F)

I can’t imagine there being (rules) because we’re all kind of adults now so I expect the rules, you know, just stay within the law, I assume. (S21, M)

Students were shown the alcohol awareness webpages described above and asked (a) whether they were familiar and (b) what they thought of content. Despite being signposted to these at the start of the year, most did not recall seeing them, citing being ‘*swamped at the beginning*’ (S23, M). When asked to reflect on content, the majority suggested that such messaging had been seen before and was therefore not needed at this age. It was also suggested that advice on strategies to limit consumption were unrealistic, particularly during early stages of university life where the social benefits of drinking with new peers outweighed any concerns over potential alcohol harms:

Alcohol probably does make it easier, takes away the awkwardness of it I think. You can have something in common when you’re drinking with people. (S2, M)

When asked what the university should do about student drinking, responses reflected the growing emphasis on personal responsibility associated with the young adult life-stage (Arnett, 2004). Most placed the onus of responsibility for drinking choices – and drinking outcomes – on themselves:

The thing is, like, I think really we should be doing it ourselves because we’re all adults so we should…if we get ourselves into a situation it’s our fault really isn’t it? (S18, F)

Students commonly felt that, as adults, learning to drink more sensibly is best achieved through personal experience rather than instruction or advice, resulting in resistance to alcohol guidance from the university:

Even if someone gave me advice I probably would still do it so I think you kind of need to learn from your own mistakes as well. (S8, F)

Like the best way of finding things out is actually doing things myself. I’ve been there and I’ve been in those speeches and just been like ‘oh I can’t wait to go…’ (S16, M)

As with staff, students were asked their opinion of more interventionist approaches, including sanctions and limits to sales or consumption. Such measures were perceived by some respondents as unfair on those who had ‘learned’ effectively in relation to drinking, with problematic use relegated to a minority behavior among those who had not yet acquired the capacity for sensible consumption:

Personally I think you need to learn yourselves…maybe (rules) should be put in place but then again you think, well would that ruin it for everyone else that does take it sensibly that is alright with their drink because some people have gone over the top. (S23, M)

Strong contradiction emerged between staff conceptualization of the provision of educational material and student perceptions of such measures. While staff favored this as appropriate for young adults and for the relationship between student and university, students were likely to interpret alcohol education as reminiscent of school and the treatment of younger people. There was clear rejection among a strong majority of students of the approaches already experienced in compulsory schooling:

This is like the same sort of thing that’s drummed into you at school like drugs are bad, don’t drink too much, things like that… people aren’t going to care… (S17, M)

You get taught like in school not to drink too much but…don’t know, I don’t think it would really alter many people’s behavior unless they had a real problem where they like depended on it. (S13, F)

When asked who would constitute effective messengers for alcohol content, students generally felt that neither non-academic or academic staff would be appropriate, with this again rejected as conflicting with adult status:

I don’t think any student wants to hear their lecturer or teacher talking about drinking, you know, they’re going to be sitting there like ‘oh you’re just like my parents’. (S15, F)

A majority of students, including the heaviest drinkers, do not see any need to reduce consumption levels (Roche & Watt, 1999). Although this was echoed here, students did suggest that, should alcohol awareness activity take place, it may be more effective if peer-led:

They’re at the same kind of level. If you speak to the staff it feels like…they’d be judging because they’re older. (S19, F)

Second year students I’ve spoken to they’ve been a bit more like oh yeah this club is good, or this place has got really cheap drinks… So maybe if one of them was to turn around and say be careful, you might have a bit more of an effect because you’re not expecting it of them… (S23, M)

As education was largely rejected, we discussed what would be considered as appropriate intervention from the university. Most students were in favor of harm reduction activities, such as safe transport home services, suggesting that the perceived inevitability of heavy drinking meant that pragmatic measures were more acceptable.

## Discussion

This research aimed to understand the development of on-site alcohol practices at one UK university and the reaction of students to these. Findings illustrate that alcohol was normalized in student identity and widely accepted by both staff and students. However, this acceptance was accompanied by a widely held view among staff that the university has a responsibility to try to reduce alcohol harms through education of students, which forms the basis of current practice on campus. This reflected perceptions of educational interventions as non-coercive and within appropriate boundaries of the staff–student relationship, with low acceptability of more interventionist approaches with a group engaging in a legal behavior with strong cultural associations. However, despite staff approval of educational approaches as appropriate for this audience, students rejected these same approaches as reminiscent of more child-like, school experiences. Research suggests that, where the legal drinking age is 18, students recognise their own legal drinker status as rationale for self-regulation over institutional intervention (Snow, Wallace, Staiger, & Stolz-Grobusch, 2003), leading to rejection of attempts to moderate alcohol use or behavior (Banister & Piacentini, 2008), and this was evident here.

Although staff participants strongly perceived a duty to aid students in making drinking decisions, the absence of *in loco parentis* status and the legality of consumption meant that defining what this should entail was problematic. Multiple internal influences were identified as shaping the organizational approach, include drinking expectations and modular student identities which acted to limit capacity for intervention. The overlap of multiple role identities is normalized in complex organizations (Webb, 2006), meaning staff in non-academic roles were required to here act in turn as business owners, landlords, carers and disciplinarians, with each of these necessitating different responses. Further, staff responses to student alcohol use, and the perceived importance of action, were often shaped by direct impacts of alcohol on daily function within departments. Those who were directly affected most frequently were more likely to try to formulate their own responses at departmental level, e.g. creation of alcohol educational materials, with little coordinated action across the institution.

Should an institutional-level response, e.g. campus-wide policy be attempted, the composition of the staff sample in this organization may be relevant. Here, senior staff roles were more likely to be occupied by male than female staff and, although this is relatively reflective of the construction of student advisory services in universities (Duffy, 2010), it may be significant in the development of organizational practice. Senior staff are more likely to be on committees where policies are developed and practice decisions are made, meaning that if men and women perceive the issue differently, outcomes may be impacted. Women typically express greater support for more interventionist alcohol policy than men (Li et al., 2017) but this may not be reflected in practice development where gender imbalance exists. Although no significant difference was noted here between male and female staff views of education, male staff did express objection to over-reach by the university more often, which may impact the direction of organizational responses.

Student perceptions of an appropriate organizational role in addressing alcohol use reflected the perceived right to live free from attempted constraints. Macro-cultural conceptualizations of alcohol locate it in the movement between work-time and play-time and the consequent switch of role identity (Gusfield, 1987) from employee to social self. However for students, who may live, work and play in the same place, this segmentation does not occur meaning they are likely to reject alcohol intervention from any part of the organization, as seen here. The right of UK adults to drink is culturally, and legally, embedded. In young adults this is accompanied by narratives of drinking to excess as a natural part of youthful risk taking in the search for autonomy (Jack, 1986), with drinking with peers perceived as significant to sociability (Griffin, Bengry-Howell, Hackley, Mistral, & Szmigin, 2009) and the UK student experience (Seaman & Ikegwuonu, 2010). In this research, both staff and students recognised the emerging adult status of students, signified by rejection of adolescent labels and expectation of increased choice and personal responsibility (Arnett, 2004). However, their conclusions over the implications of this for alcohol education were significantly different. Staff were likely to conclude that educational approaches were most respectful of the right of young adults to choose, representing a non-invasive, pragmatic option. However, students interpreted attempts at alcohol education as reminiscent of more invasive, school-like practices, leading to rejection and resistance to such messages.

In terms of evidence of effectiveness, staff views expressed here of education as a more respectful, ‘hands-off’ approach also suggest that attaining impact through alcohol programs may be secondary to other concerns around appropriateness and the organizational relationship with students. Evidence supporting educational approaches is weak but does suggest that most effective education programs for drug and alcohol prevention are interactive (Botvin & Griffin, 2007), which may contrast with the perceived right to self-determination strongly expressed here.

It was also evident here that alcohol awareness messages offering advice on ways to limit consumption, specifically those emphasising a choice to adopt ‘sensible’ drinking, contrasted with participant understandings of alcohol as a useful social practice, and as accepted within broader cultural conceptions of student behavior. This understanding reflects culturally sanctioned presentations of student drinking as expected and as constituting a rite of passage during this life-stage, with little evidence to suggest desire to change current behavior among students. Both participant groups reflect liberal conceptions of the right of legal adults to self-determine and the onus on personal responsibility for behavior inherent in much of public health (Minkler, 1999). This arguably overlooks more ecological influences on behavior (McLeroy, Bibeau, Steckler, & Glanz, 1988) which are significant in a marketized alcohol system which normalizes affordable, accessible consumption (Room, 2011). These wider influences, coupled with expectations and stereotypes of student drinking that normalize consumption, mean that, although moderation is promoted within educational messages, it is unlikely replace youth conceptions of alcohol as a tool for achieving drunkenness.

### Suggestions for practice

A key issue for alcohol intervention is the extent to which education could be effective in an alcohol-intense environment in a UK legislative setting where drinking is a legal adult behavior and is accepted as part of emerging adult development. Despite overall reductions in youth consumption at population level, the continued dominance of binge-style drinking suggests that challenges to associated harms may require different approaches to observed education-based models. It is argued therefore that the focus for universities should be on harm reduction practices, with potential development of peer-led programs, subject to evaluation.

Despite belief among participants that the SU should provide alcohol awareness and education, student acceptability was here low and other business requirements are also influential. Role segmentation was evident in the SU who, although often being the main alcohol retailer on site, were here expected to lead on alcohol education. This is problematic due to the economic benefits of student drinking to their continuance, particularly in the face of intense local competition for student customers. As stated, where economic and health motivations are in direct competition, economic imperatives will generally win (Jayne et al., 2008), meaning that expecting SU’s to act against their economic interests is likely unrealistic. However their desire to be recognised as responsible retailers, including implementation of server training schemes, safe transport schemes, etc., means they are positioned to effectively contribute to harm reduction.

This study illustrated greater acceptability for harm reduction over educational or interventionist approaches from a majority of participants, suggesting development of explicit harm reduction goals may be beneficial. It is argued that focus on harm reduction would involve re-defining success, e.g. aiming for reduced negative outcomes rather than reduced drinking (Logan & Marlatt, 2010). It is recommended that harm reduction practices be informed by local understanding of negative alcohol outcomes, through consultation with staff and students on site, thus adding a peer-led, more credible, element to content. Although evidence is currently limited, peer-led approaches have shown some positive outcomes in these settings (Tollison et al., 2008) and should be explored further.

### Limitations

A limitation of the study is the non-inclusion of academic staff as well as a broader range of external stakeholders across wider university and community settings. This may be particularly relevant in city-based campuses, where off-site consumption is the norm and student drinking is therefore impacted by local licensing practice as well as SU policy. While the regulatory and enforcement role of on-campus staff was included, other insights may have been overlooked. Although staff participants were identified as those most relevant for understanding institutional responses to student alcohol use on campus, it was evident that their own experiences shaped practice, meaning findings may not be relevant at other sites where these experiences vary. As this study selected one site for investigation, caution must be exercised in relating findings to other university contexts with significant variations of size, profile of institution, student demographics, etc.

Despite this, the research provides valuable insights into the difficult relationship between perceptions and organizational approaches to tackling alcohol behavior. Addressing this may be important in developing cohesive organizational strategies with high acceptability to those tasked with delivering outcomes. Research in universities of varying size and location is recommended to further enhance understanding.

## Conclusions

While staff favor alcohol education as acceptable and respectful of student status, students reject it as ineffective and reminiscent of school-based approaches. Consideration should be given to more realistic policy and intervention focus for UK universities, aimed at reduction of alcohol-related harms, through targeted interventions reflecting the range of practices, locations and populations observed in student consumption. This acknowledges the constraints to likelihood of reducing consumption through university activities alone and acknowledges the higher acceptability of harm reduction. Consideration should also be given to trialling and evaluating peer-led alcohol activities to understand their potential application in this context.
